# Effectiveness of nurse-led interventions on psycho-social aspects among adolescents

**DOI:** 10.6026/973206300210181

**Published:** 2025-02-28

**Authors:** Santhi Subramaniam, Jamunarani Perumalsamy, Jeevithan Shanmugam, Umapathy Pasupathy

**Affiliations:** 1 Department of Psychiatric Nursing, Sri Ramachandra Institute of Higher Education and Research (Deemed to be University) Porur, Chennai - 600116, Tamilnadu, India; 2 Department of Psychiatric Nursing KMCH College of Nursing, Coimbatore - 641048, Tamilnadu, India; 3 Department of Community Medicine, KMCH Institute of Health Sciences and Research, Coimbatore - 641014, Tamilnadu, India; 4 Department of Pediatric Medicine, Sri Ramachandra Medical College & Research Institute, Sri Ramachandra Institute of Higher Education and Research (DU), Porur, Chennai - 600116 Tamilnadu, India

**Keywords:** Nurse-led interventions, resilience, adolescents

## Abstract

Adolescence is a critical developmental stage marked by significant psychological and social challenges. Effective interventions during this period are essential for fostering long-term mental health. Hence, a true experimental pretest-posttest control group design involving 20 ninth-grade students from government-aided and private schools in Coimbatore, Tamil Nadu was completed. The eight-week nurse-led intervention included sessions on emotional self-awareness, stress coping, resilience building and mindfulness. The intervention group exhibited significant improvements in emotional regulation (mean increase of 9.8, p < 0.001) and resilience (mean increase of 22.6, p < 0.001) compared to the control group, which showed no significant changes. Further, a moderate positive correlation (r = 0.35, p = 0.05) was observed between gains in emotional regulation and resilience within the intervention group.

## Background:

Adolescence represents a pivotal period of growth, marked by significant psychological, emotional and social challenges. Effective interventions during this phase can shape not only immediate well-being but also long-term mental health outcomes. School nurses, as accessible and trusted professionals, are uniquely positioned to implement tailored psychosocial interventions. This introduction explores the efficacy and mechanisms of nurse-led interventions (NLIs) aimed at improving psycho-social aspects such as emotional regulation and resilience among school-aged students, drawing on recent research evidence. The mental health needs of students are increasingly prominent, with many adolescents struggling with emotional and social challenges that can adversely impact academic performance and overall well-being [1, 2]. Studies underscore the potential of psychosocial interventions in reducing these issues and promoting adaptive behaviours, particularly when implemented in school settings [3]. Resilience, defined as the capacity to adapt positively in the face of adversity, is a critical determinant of mental health. Evidence suggests that resilience-focused interventions not only mitigate psychological distress but also enhance academic performance and social competence [4]. Emotional regulation, another core component, has been linked to decreased anxiety, better peer relationships and improved decision-making [5]. School nurses play an instrumental role in promoting emotional well-being through structured, evidence-based interventions. Nurse-led resilience programs have demonstrated significant benefits in enhancing coping mechanisms and fostering emotional resilience among students [6]. This, such interventions are particularly effective when integrated into the school environment, as they allow for timely identification and support of at-risk students [7]. Programs involving emotional regulation training have consistently shown improvement in students' abilities to manage stress and adapt to challenges. These findings are reinforced by meta-analyses highlighting the sustained impact of school-based social and emotional learning programs [8, 9]. Mindfulness-based interventions led by school nurses have proven effective in reducing stress and enhancing focus among students [10]. Therefore, it is of interest to assess the effectiveness of nurse-led interventions on psycho-social aspects among adolescents.

## Methodology:

## Research design:

A true experimental design with pretest-posttest control groups was used. Students were randomly assigned to either an intervention (study) or control group.

## Setting:

The study was conducted in selected government-aided and private schools in Coimbatore, Tamil Nadu.

## Participants and sampling:

A total of 20 ninth-grade students were recruited. Ten students were assigned to each group using cluster random sampling. Inclusion criteria included low resilience and emotional regulation scores.

## Intervention:

The intervention included Nurse-Led Interventions (NLI) delivered over eight weeks. Sessions focused on emotional self-awareness, stress coping, resilience and mindfulness using role-play, discussions and videos.

## Data collection tools:

The Emotional Regulation Questionnaire (ERQ-CA) and Bharathiar University Resilience Scale measured emotional regulation and resilience, respectively.

## Data collection procedure:

A pre-test was conducted for both groups. After the eight-week intervention for the study group, a post-test was conducted for both groups.

## Data analysis:

Descriptive statistics summarized data. Paired t-tests analyzed within-group differences and independent t-tests compared groups. Correlation analysis examined relationships between variables.

## Ethical considerations:

Ethical approval, parental consent and student assent were obtained. Anonymity and confidentiality were maintained.

## Results:

Table 1 showed significant improvements in the study group, with emotional regulation shifting from 30% low and 70% moderate to 60% moderate and 40% high post-intervention, while the control group showed minimal change. Resilience levels also improved, with a shift from 90% moderate to 70% high in the study group, unlike the control group, which remained largely unchanged. Table 2 showed emotional regulation increased from 21.8 (SD = 4.34) to 31.6 (SD = 5.62), and resilience rose from 83.7 (SD = 7.54) to 106.3 (SD = 8.84), both with p < 0.001, while the control group showed no significant change. Table 3 revealed a moderate positive association (r = 0.35, p = 0.05) between emotional regulation and resilience gains, suggesting that improvements in one contributed to enhancements in the other.

## Discussion:

The results of this pilot study indicate that structured nurse-led interventions (NLIs) significantly enhance the psycho-social well-being of adolescents, particularly in the areas of emotional regulation and resilience. Specifically, the intervention group demonstrated substantial improvements in emotional regulation scores, increasing from a mean of 21.8 (SD = 4.34) at pre-test to 31.6 (SD = 5.62) post-test (p < 0.001), compared to the control group, which showed no significant change. Similarly, resilience scores in the intervention group rose markedly from 83.7 (SD = 7.54) to 106.3 (SD = 8.84) (p < 0.001), while the control group exhibited minimal improvement. These findings are consistent with existing literature supporting the efficacy of nurse-led interventions in fostering emotional and psychological strengths among adolescents. For instance, Alodhialah *et al.* (2024) demonstrated that nurse-led resilience programs significantly enhance coping mechanisms and emotional resilience [6].

Eva *et al.* (2017) found that mindfulness-based interventions led by nurses improved self-esteem and reduced perceived stress among marginalized youth [11]. Tang *et al.* (2014) further supported these outcomes by showing that meditation retreats facilitated by nurses resulted in significant improvements in emotional intelligence and self-regulation among adolescents [12]. Michelangelo (2015) and Raghubir (2018) highlighted the positive impact of nurse-led programs on emotional intelligence in adolescents, emphasizing the role of nurses in developing these critical skills [13-14]. These studies collectively reinforce the potential of NLIs as effective tools in adolescent mental health promotion. Bernier's longitudinal study [15] further corroborates these outcomes, highlighting the sustained positive impact of nurse-led resilience interventions on adolescent well-being over time. These improvements suggest that NLIs not only address immediate psycho-social challenges but also contribute to long-term mental health and adaptive capacities. An intriguing aspect of our study is the moderate positive correlation (r = 0.35, p = 0.05) between gains in emotional regulation and resilience within the intervention group. This relationship echoes the findings of Surzykiewicz *et al.* (2022) [16], who posited that enhancements in emotional skills facilitate the development of resilience in adolescents. Additionally, Doras *et al.* (2023) [17] emphasized the interconnectedness of emotional regulation and resilience, advocating for holistic approaches in psychosocial interventions. Our findings align with existing research on nurse-led interventions (NLIs) in improving psycho-social well-being. Pandarakutty *et al.* (2019) showed NLIs significantly enhanced HRQOL in children with sickle cell disease, similar to our study's impact on emotional regulation and resilience [18].

Huang *et al.* (2025) found NLIs improved quality of life and reduced depression in people with HIV, reinforcing their mental health benefits [19]. Varghese (2020) demonstrated NLIs effectively addressed psychosocial issues in institutionalized elderly [20], while Mathew *et al.* (2020) reported reduced problematic internet use and better biopsychosocial functioning in adolescents [21]. Like these studies, our research highlights the vital role of NLIs in fostering resilience and adaptive coping in adolescents. Our study supports this perspective, suggesting that interventions targeting emotional regulation may inherently bolster resilience, thereby amplifying the overall effectiveness of NLIs. Despite these promising results, the pilot nature of the study entails certain limitations. The small sample size (n=20) restricts the generalizability of the findings and underscores the need for larger-scale studies to validate these outcomes. The short duration of the intervention (eight weeks) may not capture the long-term sustainability of the observed benefits. Future research should consider longitudinal designs to assess the enduring effects of NLIs and explore the mechanisms underlying the interplay between emotional regulation and resilience. In conclusion, this pilot study contributes to the growing body of evidence supporting the effectiveness of structured nurse-led interventions in enhancing emotional regulation and resilience among adolescents. By fostering these critical psycho-social competencies, nurses can play a pivotal role in promoting the holistic development and mental well-being of students, thereby creating a more supportive and resilient school environment. The significant improvements observed in emotional regulation and resilience among adolescents underscore the value of integrating NLIs into school health programs. School nurses, equipped with the necessary training, can implement evidence-based interventions that address the unique psycho-social needs of students. This proactive approach not only mitigates current emotional and social challenges but also equips adolescents with the skills needed to navigate future adversities effectively.

## Recommendations for future research:

Future studies should aim to replicate these findings with larger and more diverse samples to enhance generalizability. Additionally, exploring the long-term effects of NLIs through longitudinal studies would provide deeper insights into the sustainability of the benefits observed. Investigating the specific components of NLIs that most effectively contribute to emotional regulation and resilience could further refine intervention strategies, ensuring maximum impact.

## Limitations:

This study's primary limitation is its small sample size, which may limit the ability to generalize the findings to a broader population. The reliance on self-reported measures could introduce response bias. Future research should incorporate objective assessments and consider a more extensive and varied participant pool to address these limitations.

## Figures and Tables

**Table 1:** Distribution of pre-test and post-test levels of emotional regulation and resilience

**Measure**	**Group**	**Low (%)**	**Moderate (%)**	**High (%)**
Emotional Regulation	Pre-Test Study	30%	70%	0%
	Pre-Test Control	30%	70%	0%
	Post-Test Study	0%	60%	40%
	Post-Test Control	20%	80%	0%
Resilience	Pre-Test Study	0%	90%	10%
	Pre-Test Control	0%	80%	20%
	Post-Test Study	0%	30%	70%
	Post-Test Control	0%	80%	20%

**Table 2:** Comparison of mean emotional regulation and resilience scores

**Measure**	**Group**	**Pre-Test Mean (SD)**	**Post-Test Mean (SD)**	**Difference**	**Significance**
Emotional Regulation	Study Group	21.8 (4.34)	31.6 (5.62)	9.8	p < 0.001
	Control Group	22.5 (5.25)	23.3 (5.27)	0.8	Not Significant
Resilience	Study Group	83.7 (7.54)	106.3 (8.84)	22.6	p < 0.001
	Control Group	84.8 (8.43)	86.9 (7.99)	2.1	Not Significant

**Table 3:** Correlation between emotional regulation and resilience gain scores

**Group**	**Measure**	**Correlation (r)**	**Significance**
Study Group	Emotional Regulation & Resilience Gains	0.35	p = 0.05
Control Group	Emotional Regulation & Resilience Gains	0.17	Not Significant
